# Efficacy of lenvatinib combined with sequential transarterial chemoembolization for primary hepatocellular carcinoma and the effects on serum basic fibroblast growth factor and vascular endothelial growth factor

**DOI:** 10.3389/fphar.2022.965770

**Published:** 2022-10-21

**Authors:** Qing-Yun Xie, Lu-Ping Huang, Feng-Wei Gao, Da-Qing Liu, Xia Wang, Kang-Yi Jiang, Jie Gong, Xin Zhao, Ben-Jian Gao, Ze-Hua Lei

**Affiliations:** ^1^ Department of Hepatobiliary Pancreatic Surgery, The People’s Hospital of Leshan, Leshan, China; ^2^ Department of Nuclear Medicine, The People’s Hospital of Leshan, Leshan, China

**Keywords:** hepatocellular carcinoma, transarterial chemoembolization, lenvatinib, basic fibroblast growth factor, vascular endothelial growth factor

## Abstract

**Objective:** The aim of this research was to investigate the therapeutic efficacy of lenvatinib combined with sequential transarterial chemoembolization (TACE) on primary hepatocellular carcinoma (HCC) and the effects on serum basic fibroblast growth factor (bFGF) and vascular endothelial growth factor (VEGF).

**Method:** A total of 104 patients with primary HCC, admitted to People’s Hospital of Leshan from April 2018 to January 2021, were selected as the study subjects and were divided into the TACE-LEN group (n = 53) who were treated with lenvatinib combined with sequential TACE and the TACE group (n = 51) who were treated with TACE alone, according to the appropriate treatment modalities. The clinical efficacy 8 weeks after treatment; the serum levels of total bilirubin, conjugated bilirubin, and alanine aminotransferase (ALT); the prothrombin time (PT); the indocyanine green retention rate at 15 min (ICGR15); and the serum bFGF and VEGF levels before treatment and at 8 weeks after treatment were compared between the two groups. The incidence of adverse events and the survival rates at 18 months were also recorded for both groups. COX regression analysis was used to analyze the risk factors affecting the survival of patients.

**Results:** Eight weeks after treatment, the objective response rate was higher in the TACE-LEN group than in the TACE group (77.36% vs. 56.36%, *p* < 0.05), but there were no statistically significant differences in the bilirubin and ALT levels, the PT, and the ICGR15 between the two groups (*p* > 0.05). The serum bFGF and VEGF levels post-therapeutic were lower in the TACE-LEN group than in the TACE group (*p* < 0.05). The differences in the incidence of postoperative adverse events and the survival rate within 6 months were not statistically significant between the two groups (*p* > 0.05). In addition, the survival rates within 12 and 18 months after treatment were higher in the TACE-LEN group than in the TACE group than in the TACE group (81.1% vs. 64.7%, 69.8% vs. 49.1%, *p* < 0.05). ICG-R15 and treatment regimen are risk factors for survival.

**Conclusion:** The worse the liver reserve is, the worse the prognosis is. The combination of TACE and lenvatinib showed better efficacy and longer survival than TACE monotherapy for HCC patients and reduced the levels of bFGF and VEGF.

## 1 Introduction

Hepatocellular carcinoma (HCC) is a malignant tumor with high incidence and mortality rates in China. Relevant studies (Berretta et al., 2019; Liu et al., 2022) have found that basic fibroblast growth factor (bFGF) and vascular endothelial growth factor (VEGF) are body-specific angiogenic factors that can create favorable conditions for the invasion and metastasis of cancer cells, and both bFGF and VEGF are involved in the progression of HCC. Surgery is currently the primary treatment for primary HCC, but transarterial chemoembolization (TACE) has become the first choice for patients with HCC who cannot undergo radical surgical resection as it can effectively control tumor growth, improve patient prognosis, and has the advantages of repeatability, low trauma, and few surgical complications. (Garg et al., 2022). However, the efficacy of TACE is easily influenced by factors such as the arterial source of the blood supply and the location of the tumor, especially in mid-to-late-stage primary HCC that occurs in specific locations. Additionally, complete necrosis of the tumor cells is often difficult to achieve with TACE alone. In addition, TACE treatment alone may promote tumor revascularization and stimulate the secretion of necrotic tissue, both of which increase the risk of late recurrence. (Liu et al., 2021; Zhang et al., 2022). Lenvatinib, an oral multikinase inhibitor, is an extremely potent angiogenetic inhibitor that has been shown to inhibit tumor proliferation signaling by inhibiting VEGF receptor-one to three, FGF receptor one to four, and platelet-derived growth factor receptor *α*. (Marzi et al., 2022; Ouyang et al., 2022). In addition, lenvatinib may normalize tumor vasculature, thereby facilitating the distribution and delivery of anticancer drugs. (Young et al., 2022). Currently, several prospective studies have demonstrated good therapeutic efficacy of lenvatinib in combination with TACE for the treatment of HCC, especially in cases with portal vein tumor thrombosis. (Ding et al., 2021; Yang et al., 2021). In this current study, a comparative analysis of the efficacy and post-therapeutic changes in the serum levels of bFGF and VEGF in 53 patients with primary HCC treated with lenvatinib combined with sequential TACE and 51 patients treated with TACE alone was retrospectively conducted.

## 2 Material and methods

### 2.1 General material

A total of 104 patients with primary HCC, admitted to People’s Hospital of Leshan from April 2018 to January 2021, were selected as the study subjects. They were divided into the TACE-LEN group (n = 53), who were treated with lenvatinib combined with sequential TACE, and the TACE group (n = 51), who were treated with TACE alone, according to the appropriate treatment modalities.

The inclusion criteria were as follows: (1) HCC diagnosed clinically or by pathological results, and clinical stage was BCLC stage B or C; (2) previously treated with TACE alone or TACE in combination with lenvatinib; (3) no history of antitumor therapy in the 6 months preceding TACE treatment; (4) a Child–Pugh score for hepatic function of A or B; (5) Eastern Cooperative Oncology Group (ECOG) score of ≤2 points; (6) normal cardiac and renal function; (7) conscious and communicative; (8) expected survival of more than 3 months; and (9) complete clinical data available for analysis.

The exclusion criteria were as follows: (1) aged <18 or ≥75 years; (2) rupture and hemorrhage due to esophageal varices within the preceding 3 months; (3) the use of systemic antineoplastic drugs; (4) pregnancy; (5) hepatic artery thrombosis; (6) acute tumor rupture combined with hemoperitoneum; (7) extrahepatic organ metastases, excluding local lymph node metastases; (8) a history of hepatic encephalopathy; (9) heart failure or combined coronary artery disease; or (10) malignant tumors of other organs.

### 2.2 Surgical methods

#### 2.2.1 The TACE group

The Seldinger technique for modified vascular puncture was conducted on the root of the thigh in each patient. After local anesthesia, a 5F catheter sheath was inserted and an angiographic catheter (5F RH, TERUMO Corporation, Japan) was inserted into the celiac trunk or superior mesenteric artery to confirm the blood supply to the tumor. Then, the microcatheter (2.5F Cantata^®^ Microcatheter, Cook Medical, United States) was super-selectively inserted into the blood supply vessels of the HCC tissue, and the embolic agent was injected into the target artery to block the blood supply to the tumor tissue and release the chemotherapeutics to continuously attack the tumor. If necessary, blank microspheres (Embosphere Microspheres 100–300 μm, Merit Medical Systems, Inc., United States) were adopted as a supplemental bolus. The endpoints of embolization were as follows: visualization of the small branches of the tumor portal vein, stagnation or regurgitation of the embolic agent at the tip of the catheter, disappearance of tumor staining on postoperative imaging, and preservation of the main vessels. For huge HCCs, depending on the liver reserve function of the patient, two TACE treatments at 2-week intervals were conducted to achieve the embolization endpoints if necessary. Subsequently, a computed tomography (CT) or magnetic resonance image (MRI) review was performed every 6–8 weeks, and the subsequent TACE treatment was conducted as needed, depending on the residual active tumor. The TACE therapy was ended if the following conditions were met: (1) complete obstruction with major large vessel infiltration, (2) no active target foci on image evaluation, (3) disease progression evaluated by two successive images according to the Modified Response Evaluation Criteria in Solid Tumors (mRECIST) (Ding et al., 2021) therapeutic evaluation, or (4) long-term hepatic impairment (a Child–Pugh score ≥9 points for more than 4 weeks).

Note: There were two options for the preparation of the embolic agents: (1) drug-eluting microspheres (HepaSphere™ Microspheres 50–100 μm, Merit Medical System, Inc., United States) loaded with 60–80 mg of epirubicin (Pfizer, United States) or (2) emulsifier prepared with 5–20 ml of Lipiodol (Lipiodol^®^ Ultra Fluid, Guerbet, France) with 60–80 mg of epirubicin.

#### 2.2.2 The TACE-LEN group

The initiation process and termination process for TACE were the same as in the TACE group.

The lenvatinib (Eisai Co., Ltd., Japan) therapy was started once the patient was clinically diagnosed with unresectable HCC at a dose of 8 mg orally once per day (bodyweight <60 kg) or 12 mg orally once per day (bodyweight ≥60 kg). Lenvatinib was started 3 days after the first TACE and then stopped within 3 days before or after each TACE. The start of the observation time is the date of TACE.

#### 2.2.3 Postoperative management

Postoperatively, the patients in both groups were given routine symptomatic treatment, such as liver protection and pain relief. Liver and kidney function, routine blood tests, coagulation function, and a review of serum bFGF and VEGF levels were conducted 8 weeks postoperatively. Enhanced MRI or CT scans were performed six to 8 weeks postoperatively, and the subsequent TACE treatment was performed as needed, according to the residual active tumor. The doses were adjusted according to the grade of the National Cancer Institute’s Common Terminology Criteria for Adverse Events version 4.0. Lenvatinib was continually administered until disease progression or the occurrence of unacceptable adverse events. All enrolled patients were followed up with for 18 months consecutively, and follow-up contact was terminated when the patient died.

### 2.3 Observation indicators

According to mRECIST (Ilagan et al., 2022) standards, the clinical outcomes (including complete response, partial response, stable disease, and disease progression) were compared between the two groups 8 weeks after treatment. The objective response rate (ORR) was calculated as the sum of the percentage of patients with complete response and with partial response. The serum levels of total bilirubin, conjugated bilirubin, and alanine aminotransferase (ALT); the prothrombin time (PT); the indocyanine green retention rate at 15 min (ICGR15); and the serum bFGF and VEGF levels before and at 8 weeks after treatment were compared between the two groups. The incidence of adverse events and the survival rates within 18 months were recorded for both groups.

### 2.4 Statistical methods

The SPSS 23.0 software was adopted for statistical processing of all the data obtained. The ORR, incidence of adverse events, and other countable data were expressed as rates (%), and the chi-squared (χ^2^) test was used for comparison. The liver function, bFGF and VEGF levels, and other measurement data were expressed as the mean ± standard deviation. The independent samples *t*-test was used for data comparison. The Kaplan–Meier survival curves were plotted, and the log-rank test was used to compare the survival rates. A value of *p <* 0.05 was considered statistically significant.

## 3 Results

### 3.1 General characteristics

As is shown in Table 1, the differences in gender, age, preoperative liver function, and the diameter and number of the tumors at baseline were not statistically significant between the two groups (*p* > 0.05), and the data were comparable.

**TABLE 1:** Comparison of the general characteristics between the two groups of patients.

The general characteristics	TACE (n = 51)	TACE-LEN (n = 53)	*t/x* ^*2*^	*p*
Gender (Male/Female, the number of cases)	38/13	41/12	0.115^a^	0.734
The average age (Year)	56.83 ± 5.68	56.59 ± 5.74	0.214	0.831
BCLC B/C	30/21	34/19	0.312^a^	0.577
MVI(+)[%]	18 (35.3%)	16 (30.2%)	0.308^a^	0.579
Child-Pugh A/B	39/12	42/11	0.116^a^	0.733
ICG- R15 $\left(\bar{x}\pm s\right)$	11.84 ± 6.19	13.09 ± 5.23	1.114	0.268
Combined with Hepatitis B (the number of cases [%])	42 (82.4%)	45 (84.9%)	0.124	0.725
The number of TACE (Times)	2.03 ± 0.42	2.29 ± 0.95	1.793	0.076
Tumor size (cm, $\bar{x}\pm s$ )	6.56 ± 2.92	7.03 ± 3.11	0.794	0.429
Tumor number $\left(\bar{x}\pm s\right)$	2.51 ± 1.08	2.83 ± 1.34	1.338	0.184
Ascites (the number of cases [%])	8 (15.69%)	6 (11.32%)	0.425	0.514
AFP (ng/ml [the number of cases])				
<400/≥400	14/37	17/36	0.266	0.606

^a^ *x* ^*2*^-test ICG R15: the indocyanine green retention rate of 15 min.

AFP: alpha-fetoprotein. MVI(+):Microvascular invasion(positive).

### 3.2 Clinical efficacy

Table 2 shows that the ORR of the patients in the TACE-LEN group (77.36%) was significantly higher than in the TACE group (56.36%; χ^2^ = 4.962; *p* = 0.026).

**TABLE 2:** Comparison of the clinical efficacy between the two groups.

Group	Complete response	Partial response	Stable disease	Disease progression	Objective response rate
TACE-LEN (n = 53)	12 (22.64%)	29 (54.72%)	9 (16.98%)	3 (5.66%)	41 (77.36%)
TACE (n = 51)	6 (11.76%)	23 (45.10%)	12 (23.53%)	10 (19.61%)	29 (56.86%)
*χ* ^2^	—	—	—	—	4.962
*P*	—	—	—	—	0.026

### 3.3 Hepatic function

As is illustrated in Table 3, the results showed that the differences in the main indicators reflecting liver function (i.e., bilirubin, ALT, PT, and ICGR15) were not statistically significant between the two groups at 8 weeks after treatment (*p* > 0.05).

**TABLE 3:** The indicators for hepatic function 8 weeks after surgery.

The serological indicators	TACE (n = 51)	TACE-LEN (n = 53)	*t*	*p*
Total bilirubin (umol/L)	26.31 ± 7.18	29.24 ± 8.80	1.856	0.066
Conjugated bilirubin (umol/L)	11.23 ± 5.14	13.37 ± 9.36	1.437	0.154
Alanine aminotransferase (U/L)	50.17 ± 5.23	48.67 ± 4.52	1.567	0.120
Prothrombin time (S)	13.77 ± 2.65	14.02 ± 2.93	0.456	0.649
ICG R15%	12.33 ± 5.56	13.67 ± 6.82	1.096	0.276

ICG R15: The indocyanine green retention rate of 15 min.

### 3.4 Basic fibroblast growth factor and vascular endothelial growth factor levels

There was no significant difference in the levels of bFGF and VEGF between the two groups before treatment (t = 1.473, *p* = 0.144; t = 1.485, *p* = 0.141), and the levels of bFGF and VEGF in the TACE group increased after 8 weeks of treatment while those in the TACE-LEN group decreased compared with those before treatment. After 8 weeks of treatment, the levels of bFGF and VEGF in the TACE-LEN group were significantly lower than those in the TACE group, and the differences were statistically significant (*p* < 0.05). See Table 4 for details.

**TABLE 4:** Comparison of the bFGF and VEGF before and after treatment between the two groups.

The serological indicators	TACE (n = 51)		TACE-LEN (n = 53)	
	Before treatment	After treatment	Before treatment	After treatment
bFGF (pg/ml)	8.56 ± 0.81	8.98 ± 0.35	8.32 ± 0.85^&^	5.12 ± 0.55^*#^
VEGF (pg/ml)	287.74 ± 28.85	325.41 ± 24.69	279.31 ± 29.03^&^	275.12 ± 27.69^*#^

Note: &Compared with control group before treatment, *p* > 0.05; *Compared with the same group before treatment, *p* < 0.05; #Compared with the control group 8 weeks after treatment, *p* < 0.05.

bFGF: basic fibroblast growth factor. VEGF: vascular endothelial growth factor.

### 3.5 Post-therapeutic adverse events

There were no treatment-related deaths in either group after treatment. The incidence of adverse events was higher in the TACE-LEN group, but the difference was not statistically significant (*p* > 0.05). The difference in the incidence of grade 3–4 serious adverse events was not statistically significant between the two groups (*p* > 0.05). The most common adverse reaction in the TACE-LEN group was hand–foot syndrome (11.32%; see Table 5). In the TACE-LEN and TACE groups there were two cases of liver abscess adverse events in each, including two cases of patients with adverse events that scored a level 3 and a level 4 for concurrent septic shock. These patients were all treated timely with percutaneous liver puncture drainage, antibacterial drug administration, and reasonable anti-shock treatment. After treatment, the patients became stable, and neither patient had a recurrence during follow-up care. Therefore, it was determined that liver abscesses did not affect the prognosis of the patients and their long-term liver reserve function. In addition, there were two cases of cholecystitis after TACE in the TACE-LEN group, both of which were caused by ectopic embolization of a small amount of embolic agent after TACE. Both patients improved after symptomatic treatment. There were no clinical symptoms related to cholecystitis in the follow-up process, and the liver reserve function and prognosis of the patients were not affected.

**TABLE 5:** Comparison of adverse events after treatment between the two groups.

The types of adverse events	TACE (n = 51)			TACE-LEN (n = 53)		
	The total number of cases	The number of grade 3 adverse events	The number of grade 4 adverse events	The total number of cases	The number of grade 3 adverse events	The number of grade 4 adverse events
Liver abscess	2	1	1	2	1	0
Hand-foot syndrome	0	0	0	6	0	0
Hypertension	1	0	0	5	1	0
Acute liver failure	2	1	0	2	1	0
Cholecystitis	2	0	0	2	0	0
Mouth ulcers	4	1	0	3	0	0
Gastrointestinal hemorrhage	4	0	0	4	1	1
Total (%)	15 (29.4%)	3 (5.88%)	1 (1.96%)	24 (45.3%)	4 (7.55%)	1 (1.89%)

### 3.6 Post-therapeutic survival

**FIGURE 1:**
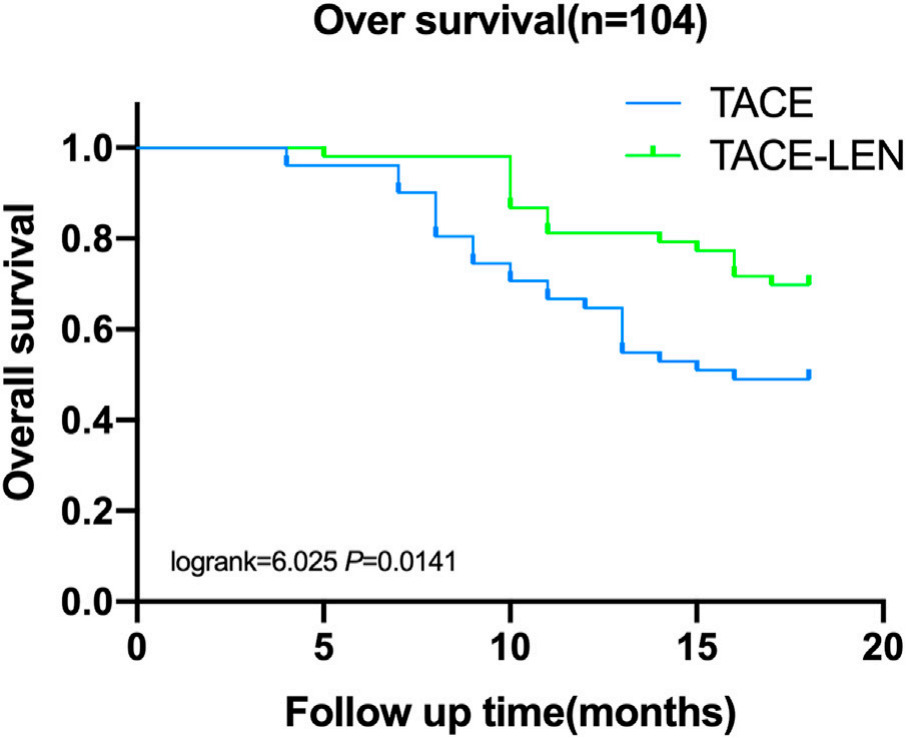
Survival proportions- Survival of Data.

Six months after treatment, the survival rate was 98.1% in the TACE-LEN group and 96.1% in the TACE group, and the difference was not statistically significant between the two groups. The survival rates 12 and 18 months after surgery were higher in the TACE-LEN group (81.1% and 69.8%, respectively) than in the TACE group (64.7% and 49.1%, respectively; log-rank = 4.409 and 6.025, respectively, *p* < 0.05, see Figure 1).

### 3.7 COX proportional hazard analysis

**FIGURE 2:**
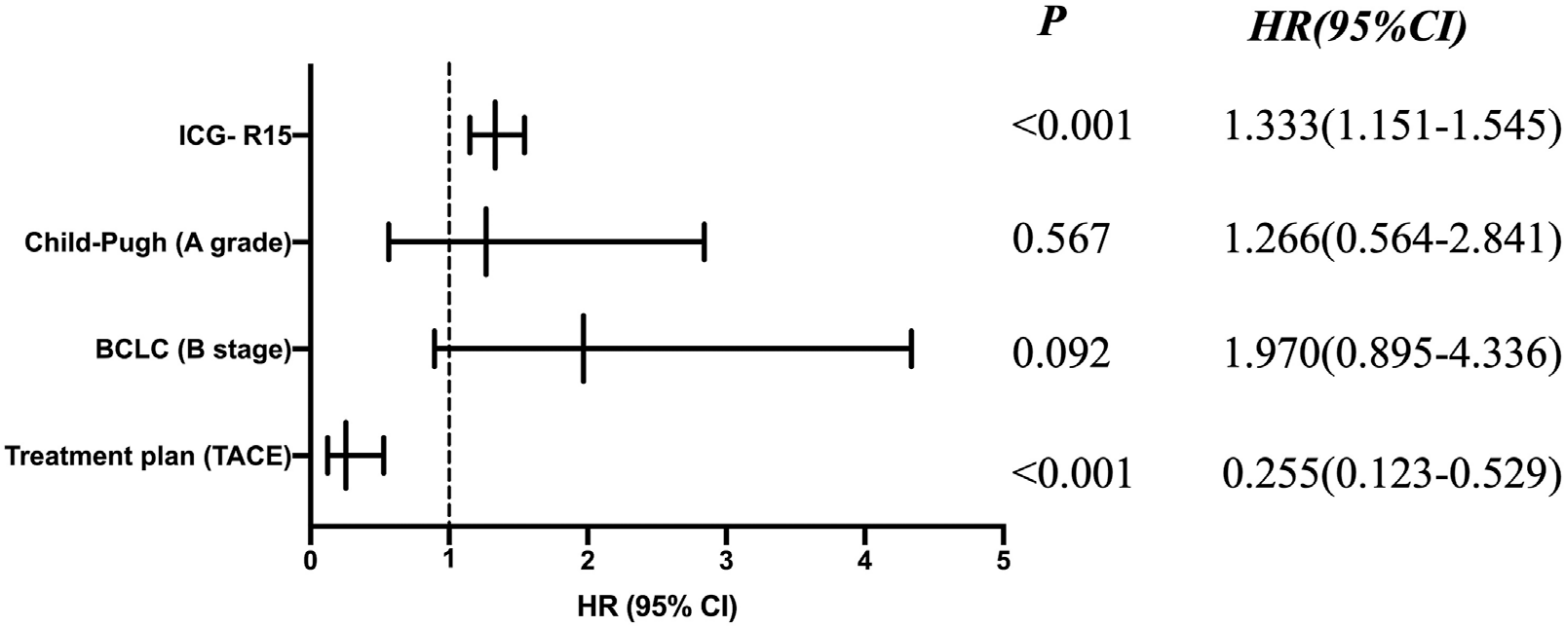
COX proportional hazard analysis for OS.

Univariate Cox regression analysis showed that treatment plan, BCLC stage, Child-Pugh classification of liver function, ICG-R15 and other factors were related to prognosis (see Table 6). Moreover, according to the multivariate COX regression analysis model, ICG-R15 and treatment regimens were independent risk factors for prognosis of patients (see Figure 2).

**TABLE 6:** Unvariate COX regression analysis affecting prognosis.

Factor	*B*	*SE*	Wald *x* ^*2*^	*p*	*HR(95%CI)*
Treatment plan (TACE)	0.748	*0.318*	5.518	*0.019*	*2.112(1.132–3.942)*
Gender (Male)	0.234	*0.441*	0.304	*0.582*	*1.275(0.537–3.028)*
Age	−0.027	0.023	1.363	0.243	0.973 (0.930–1.019)
BCLC (B stage)	−0.996	0.165	34.471	<0.001	0.380 (0.276–0.525)
MVI(+)	0.408	0.351	1.351	0.245	1.504 (0.756–2.993)
Child-Pugh (A grade)	−1.671	0.331	27.040	<0.001	0.188 (0.100–0.353)
ICG- R15	0.286	0.046	38.755	<0.001	1.331 (1.216–1.456)
Combined with Hepatitis B	−0.875	0.526	2.767	0.096	0.417 (0.149–1.169)
The number of TACE	−0.478	0.286	2.789	0.095	0.620 (0.354–1.087)
Tumor size	−0.010	0.101	0.010	0.920	0.990 (0.813–1.206)
Tumor number	−0.220	0.152	2.099	0.147	0.802 (0.596–1.081)
AFP(≥400 ng/ml)	0.309	0.322	0.920	0.337	1.362 (0.724–2.561)

B: regression coefficient and intercept, SE: standard error, Wald X2: wald chi-square, P: p-value, prabability. HR: hazard ratio, 95% CI: confidence interval.

## 4 Discussion

Primary HCC has high morbidity and mortality rates and is mainly treated with surgical resection, liver transplantation, TACE, biological therapy, systemic chemotherapy, and hormone therapy. Surgery is the most common therapeutic method for HCC, but some patients cannot tolerate surgery due to factors concerning the site and size of the tumor, underlying liver lesions, hepatic reserve function, and systemic diseases. (Fu and Wang, 2018). Transarterial chemoembolization is a method in which chemotherapeutics are injected through the blood supply arteries to cause death and necrosis in the tumor cells; it is simple, non-invasive, and has a high rate of short-term remission. It can also block the blood supply to the tumor at the conduction site of the chemotherapy to achieve the therapeutic purpose. Therefore, it is the first choice for non-surgical treatment of primary HCC. (Ziogas et al., 2017).

Mutsuki et al. (Matsuki et al., 2018) conducted an experiment using lenvatinib on nine types of HCC and showed that lenvatinib may bind to VEGF2 and fibroblast growth factor receptor one in a V-binding model as well as block the phosphorylation of the downstream molecules, thus exerting a powerful anti-angiogenic effect that inhibits tumor growth. An open, multicenter, phase III non-inferiority trial of lenvatinib *versus* sorafenib conducted by Kudo M. (Kudo et al., 2018). showed that lenvatinib was not inferior to sorafenib in terms of antitumor activity. In terms of the secondary efficacy endpoints, such as progression-free survival (PFS), time to progression, and ORR, lenvatinib was significantly superior. Therefore, lenvatinib is now classified as the first-line recommended drug for the systemic treatment of mid-to-late-stage HCC in China.

However, TACE alone may cause incomplete tumor embolization, resulting in hepatic function impairment and adverse events such as stimulating the expression of VEGF in the residual lesions, which may promote tumor vascular regeneration and lead to tumor recurrence. (Ako et al., 2018; Liu et al., 2021). Adriana et al. (Chen et al., 2022) confirmed that TACE may lead to a reduced blood supply of micro-vessels in the tumor tissue of HCC cells due to the embolic effect, causing the oxygen supply chain required for tumor growth to be cut off. When the tissue is in an ischemic and hypoxic state, VEGF and bFGF may be released and expressed in large quantities to promote the survival of neovascularization and maintain the requirements for tumor growth. Therefore, VEGF may be the most reliable prognostic parameter to be adopted as an index to evaluate the efficacy of TACE. In this study, the levels of bFGF and VEGF in the TACE-LEN group at 8 weeks after the operation were significantly lower than those in the TACE group before treatment and at 8 weeks after operation. This confirmed the clinical value of the above-mentioned studies. Therefore, it can be predicted from the mechanism of action that the anti-angiogenic effect of lenvatinib can effectively antagonize the high expression of VEGF after TACE, and the sequential application of the two therapeutic regimens could certainly be a new direction in the treatment exploration for HCC.

In a prospective proof-of-concept study, Masatoshi et al. (Kudo et al., 2019) compared 30 patients treated with lenvatinib in combination with TACE and 60 patients treated with TACE alone. The results showed that the hepatic function reserve was irreversibly reduced in the TACE only group, while the hepatic function was maintained at the end of treatment in the lenvatinib combined with sequential TACE group. The PFS in the lenvatinib group (16 months) was significantly longer than in the TACE only group (3 months; hazard ratio [HR] = 0.19, 95% confidence interval [CI] = 0.10–0.35). The overall survival in the lenvatinib group (37.9 months) was also significantly longer than in the TACE only group (21.3 months; HR = 0.48, 95% CI = 0.16–0.79). The study highlighted the importance of lenvatinib and that the lenvatinib combined with sequential TACE regimen may hold great promise for patients with HCC. Studies (Kudo, 2019; Young et al., 2022) have shown that the application of lenvatinib before TACE treatment may normalize the tumor vasculature, thus improving the efficacy of the intraoperative chemotherapeutic delivery in TACE, and the continued application of lenvatinib after surgery may inhibit the expression of VEGF and angiopoietin-2, thus synergistically enhancing the therapeutic efficacy of TACE. In this current study, the ORR of patients in the TACE-LEN group (77.36%) was significantly higher than in the TACE group (56.86%), and the survival rate at 12 and 18 months were also significantly higher in the TACE-LEN group than in the TACE group. These results support the findings reported above and fully reflect the advantages of lenvatinib combined with sequential TACE treatment. In clinical practice, ICG-R15 is mostly used as the detection of liver reserve function before hepatectomy and provides decision-making for the choice of surgical plan (Haegele et al., 2016). However, there are relatively few studies on the impact of ICG-R15 level before treatment on the survival and prognosis of patients. The analysis of ICG-R15 in this study shows that ICG-R15 is one of the main risk factors affecting the survival and prognosis of patients. The worse the liver reserve function is, the worse the survival prognosis is, which has certain reference value for the evaluation and prognosis of non-surgical treatment.

In terms of adverse events, there were no treatment-related deaths in either group. There was a higher incidence of adverse events in the TACE-LEN group than in the TACE group, but the difference was not statistically significant (*p* > 0.05). The difference in the incidence of grade 3–4 serious adverse events was not statistically significant between the two groups (*p* > 0.05). The most common adverse events in the TACE-LEN group were hand–foot syndrome (11.32%) and hypertension (9.43%), both of which resolved after appropriate symptomatic management. These results support the findings of previous similar studies. (Kudo, 2019). In addition, Rimassa et al. (Rimassa et al., 2019) analyzed the occurrence of various adverse events in patients taking lenvatinib by collecting data from relevant trials and confirmed that the adverse events could be prevented and effectively managed with the adoption of appropriate measures, thus improving the prognosis, overall survival, and quality of life of patients with HCC. This evidence suggests that the overall tolerability of lenvatinib combined with sequential TACE therapy is good, and the adverse effects are manageable. Therefore, the safety of the treatment is guaranteed.

In conclusion, the combination of TACE and lenvatinib was more effective than TACE alone in the treatment of patients with HCC; the combination treatment reduced the levels of bFGF and VEGF and improved the survival rate of the patients. ICG-R15 also has a certain reference value for the prognosis of non-surgical treatment, which is worthy of further clinical research and application.However, this study is a retrospective single-center cohort study with relatively short follow-up time, which is its limitation. Therefore, a large sample size, multicenter and prospective study is still needed to further confirm the safety and efficacy of lenvatinib sequential TACE regimen and expand the population benefiting from treatment.

## Data Availability

The original contributions presented in the study are included in the article/supplementary material, further inquiries can be directed to the corresponding author.
